# Factors Affecting the Age in Normal Menopause and frequency of Menopausal Symptoms in Northern Iran

**Published:** 2011-03-01

**Authors:** M A Delavar, M Hajiahmadi

**Affiliations:** 1 Department of Midwifery, Fatemezahra Infertility and Reproductive Health Research Center, Babol University of Medical Sciences, Babol, Iran; 2 Department of Social Medicine and Health, Babol University of Medical Sciences, Babol, Iran

**Keywords:** Menopause, Hot flushes, Women health, Iran

## Abstract

**Background:**

The age in menopause and frequency of menopausal symptoms differ in different societies. This study evaluates the distribution of age at normal menopause, the frequency of menopausal symptoms and the associated factors in Babol, Northern Iran.

**Methods:**

In a community based study, the Symptom Score Card was used to assess the frequency and severity of menopausal symptoms. Data from 1,397 women around 45-63 years old in Babol, northern Iran were enrolled. Subjects were selected using the standard cluster sampling techniques.

**Results:**

In urban areas, the median age at menopause was 48 years. The five most prevalent symptoms were irritabilities (72.1%), joint pains (70.6%), backache (61.2%), hot flushes (49.3%) and headache (49.2%) during the previous two weeks. More than 60% of women experienced hot flushes. Low educational level (OR=0.70; 95% CI= 0.54, 0.90), early age at menarche (OR=0.76; 95% CI=0.59, 0.99) and oral contraceptive use (OR=0.76; 95% CI=0.54, 0.97) were significantly associated with hot flushes.

**Conclusion:**

It could be beneficial to provide evidences to manage menopausal women in Primary Health Care centers as a cost effective method. Emphasis should be placed on practical education of women to raise their knowledge on menopausal symptoms and ways of increasing their understanding on midlife experiences.

## Introduction

Life expectancy has increased worldwide over the past century and most men and women are now living beyond the age of 80 years in most parts of world.[1] However, the average age of menopause still remains around 50 years.[2] Symptoms of menopause could be classified into mild, moderate or severe, with the classic symptom of hot flushes. Menopause can effect the women's quality life due to osteoporosis and cardiovascular diseases.[3] Numerous studies reported cardiovascular diseases and osteoporosis to be more prevalent in early menopaus [4][5] and the association of breast and endometrial cancers with delayed menopause.[6] The age in menopause may vary from one population to another. A number of factors can effect the age of menopause. This study aimed to determine the distribution of age in normal menopause and the associated factors.

## Materials and Methods

In a retrospective and descriptive study using standard cluster sampling technique, 1,620 women aged between 45-63 years old in Babol, northern Iran were enrolled. Sixty clusters in Babol considering the geographical area were selected (out of the 100 small administrative units) according to the latest census of the country. The subjects in our study were from the urban and peri urban areas of the city. Twenty seven women aged 45-63 years old entered the study from each cluster. The first house in the cluster was randomly selected. The second house in the cluster was next to the first house. This process was repeated until the twenty seven women in each cluster was identified and interviewed. An informed written consent was obtained from all subjects participating in the study. The women with serious illnesses (any malignancy) and history of hysterectomy or other procedures that would have stopped their menses were excluded.

A face to face household interview was conducted using a defined questionnaire. The interview was conducted by trained skilled personnel on menopausal status that lasted approximately 15 minutes. The questionnaire included menopausal symptoms, menopausal status, causes of menopause, the use of hormones for contraceptive purposes and hormone replacement therapy, reproductive history (age at menarche, gravidity, parity, abortion, age at first live birth, and pattern of menstrual cessation) and socio-demographic factors such as age, marital status, education, occupation, and economical status.

The menopausal status was assessed in a gynecological interview. The date of menopause was defined as natural cessation of menstrual period for twelve or more months.[7] For the women who had undergone hysterectomy and oophorectomy, the age of menopause was the date of surgical procedure. The women reported whether they had experienced hot flushes at the time of menopause. The Symptom Score Card was used to assess the frequency and severity of menopausal symptoms. With regards to menopausal symptoms, the women were asked if they had experienced 20 symptoms in the last two weeks and graded its severity as mild, moderate or sever.

The weight of the subjects was recorded using digital scales approximately 100 grams, with minimal clothes and without wearing shoes. The height was measured barefoot, with a tape measure.[8] The body mass index was calculated using the formula [BMI=weight (kg)/ height(2) (ml)].[9] All analyses were performed with SPSS software (Version 16.0, Chicago, IL, USA). The difference in the distribution of the women by tertiles of age in normal menopause for socio-demographic characteristic, reproductive history, body mass index and cigarette smoking variables were tested by mean and Chi-Square test. Measurement variables in two groups were compared using the student's t-test. The multiple logistic regressions were used to determine the effect of factors, significantly associated with hot flushes. The odds ratio (OR) were presented together with their 95% confidence intervals. The adjustments were made for independent variables including age, marital status, education, occupation, economic situation, smoking, age at menarche, parity, abortion, oral contraceptive use and body mass index.

## Results

A total of 1,620 women with a mean age of 51.7±5.4 years (range 45-63 years) were enrolled. Seven hundred and forty of 1,620 women had reached their menopausal period spontaneously and 223 (13.8%) had experienced menopause after total hysterectomy and were excluded from the study.

The mean age in menopause for the whole population was 46.6±5.6 years and the median age was 48.0 years. A premature normal menopause at age<40 years was reported in 5.9%, while at age>55 was 40.6%. Out of 1,397 women, 1,268 (90.8%) were married and 1,229 women (88.0%) had no income (housewives). The marital age among the women ranged from 9 years to 40 years with a mean age 18.7±4.9 years. The mean educational duration was 4.2±4.9 years. More than 56.7% (No=792) of the subjects had low elementary educational level. 181 (13.0%) women reported to be in debts. Only 14 subjects (1.0%) reported being current smokers. The mean age at menarche was 13.3±1.6 years. Among married women, the parity number ranged from 0 to 14 with mean of 4.3±1.9 birth. Eight hundred and thirty seven (59.9%) women did have any abortion. The only used hormone by women was oral contraceptive pill. Two hundred and eighty one (29.1%) women used contraceptive pills some time in their lives. The mean weight of the women was 71.3±12.6 kg and the median weight was 70.0 kg. The mean height was 156.4±5.7 cm. The mean body mass index was 29.2±5.0 kg.m(2).

Table 1 shows the mean age in normal menopause and their distribution according to tertiles of age in menopause in relation to socioeconomic status, reproductive history, and body mass index. No significant difference was observed for age in menopause, marital status and body mass index. The nullipar women had a significantly earlier age of menopause (45.8 years) compared with women with at least one child (47.8 years) (p=0.04). There was no relationship between marital status, education, occupation, economic situation, smoking, age at menarche, parity, abortion, oral contraceptive used and body mass index (Table 2).

**Table 1:** Distribution of study subjects according to tertiles of age at normal menopause with various factors in 45- 63 years women in Babol (No=740).

**Variable**	**Mean age at menopause**	**Age at menopause (years)^a^**			**p value**
		**<45 No (%)**	**45-50 No (%)**	**>50 No (%)**	
Total	47.7	154 (20.8)^b^	387 (52.3)	199 (26.9)	0.245
Marital status					
Married	47.8	137 (2.9)	336 (51.3)	182 (27.8)	
Single	47.2	17 (20.0)	51 (60.0)	17 (20.0)	
Education level(years)					0.628
<6	47.6	117 (21.5)	286 (52.7)	140 (25.8)	
6-11	47.6	21 (21.4)	50 (51.0)	27 (27.6)	
>12	48.5	16 (16.2)	51 (51.5)	32 (32.3)	
Occupation					0.908
House wife	47.7	137 (21.0)	341 (52.3)	174 (26.7)	
Working	47.9	17 (19.3)	46 (52.3)	25 (28.4)	
Economic situation					
With saving	48.1	21 (18.8)	57 (50.9)	34 (30.4)	
With debts	47.7	111 (21.5)	271 (52.4)	135 (26.1)	
No saving and no debt	47.7	22 (19.8)	59 (53.2)	30 (27.0)	
Smoking					0.430
Smoker	47.1	2 (20.0)	7 (70.0)	1 (10.0)	
Non-smoker	47.7	152 (20.8)	380 (52.1)	198 (27.1)	
Age at menarche					0.329
<13 years	47.4	42 (23.6)	95 (53.4)	41 (23.0)	
≥13 years	47.8	112 (19.9)	292 (52.0)	158 (28.1)	
Parity					0.124
Childless	45.8	10 (35.7)	13 (46.4)	5 (17.9)	
At least one child	47.8	144 (20.2)	374 (52.5)	194 (27.2)	
Abortion					0.536
No abort	47.6	92 (22.3)	213 (51.6)	108 (26.2)	
At least one abort	47.9	62 (19.0)	174 (53.2)	91 (27.8)	
Oral contraceptive used					0.471
Yes	47.4	45 (23.9)	95 (50.5)	48 (25.5)	
No	47.8	109 (19.7)	292 (52.9)	151 (27.4)	
Body mass index					0.904
Normal or less (<25.0)	47.6	39 (23.4)	84 (50.3)	44 (26.3)	
Overweight (25.0-29.9)	48.0	61 (19.9)	160 (52.3)	85 (27.8)	
Obese (≥30.0)	47.5	54 (20.2)	143 (53.6)	70 (26.2)	

^a^ Tertiles of age at menopause defined using the whole population

^b^ Row percentage

**Table 2:** Relationship between various factors and age of post-perimenopausal 45-63 yesrs women in Babol

**Variable**	**Age at menopause (years)**	**p value**
	**Mean±SD (No=740)**	
Marital status		0.389
Married	47.8±4.8	
Single	41.2±5.4	
Education		0.718
Less than elementary level	47.7±5.1	
At least elementary level	47.8±4.6	
Occupation		0.794
House wife	47.7±4.9	
Working	47.9±4.8	
Economic situation		0.370
With saving	48.1±4.6	
With debts	47.7±5.0	
Smoking		0.692
Smoker	47.1±3.3	
Non-smoker	47.7±4.9	
Age at menarche		0.310
<13 years	47.4±4.8	
≥13 years	47.8±4.9	
Parity		0.038
Childless	45.8±5.7	
At least one child	47.8±4.8	
Abortion		0.331
No abort	47.6±4.9	
At least one abort	47.9±4.9	
Oral contraceptive used		0.301
Yes	47.4±4.6	
No	47.8±5.0	
Body mass index		0.552
≤25 kg/m^2^	647.5±4.9	
>25kg/m^2^	47.8±4.9	

Table 3 illustrates the percentage of women aged 45-63 years and experienced any symptoms considered as moderate or severe. The three most prevalent moderate or severe symptoms were joint pains (34.4%), backache (31.1%) and sleeplessness (23.3%). The number of women who reported moderate or severe menopausal symptoms was considerably lower than those who reported mild symptoms. The five most prevalent symptoms were irritabilities (72.1%), joint pains (70.6%), backache (61.2%), hot flushes (49.3%) and headache (49.2%) during the previous two weeks.

**Table 3:** Prevalence of menopausal symptoms in 45-63 years old women in Babol (No=1397)

** Symptoms **	** Prevalence **	** Prevalence of moderate or severe symptoms **
	**No (%)**	**No (%)**
Hot flushes	689 (49.3)	250 (17.9)
Light – headed feelings	290 (20.8)	41 (2.9)
Headaches	688 (49.2)	270 (19.3)
Irritablility	1007 (72.1)	342 (24.5)
Depression	834 (59.7)	283 (20.3)
Unloved feelings	287 (20.5)	60 (4.3)
Anxiety	252 (18.0)	252 (18.0)
Mood changes	91 (6.5)	91 (6.5)
Sleeplessness	381 (27.3)	381 (27.3)
Unusual tiredness	374 (26.8)	374 (26.8)
Backache	855 (61.2)	435 (31.1)
Joint pains	986 (70.6)	480 (34.4)
Muscle pains	730 (52.3)	220 (15.7)
New facial hair	324 (23.2)	81 (5.8)
Dry skin	344 (24.6)	90 (6.4)
Crawling feeling under skin	243 (17.4)	75 (5.4)
Less sexual feelings	633 (45.3)	281 (20.1)
Dry vagina	408 (29.4)	166 (11.9)
Uncomfortable intercourse	514 (36.8)	220 (15.7)
Urinary frequency	418 (29.9)	176 (12.6)

Eight hundred and fifty and nine women (61.5%) experienced hot flushes at the time of menopause. Table 4 presents the estimated adjusted odds ratio (with 95% CI) of hot flushes and socioeconomical status, reproductive history and body mass index. Low educational level (OR=0.70; 95% CI=0.54, 0.90), early age at menarche (OR=0.76; 95% CI=0.59, 0.99) and oral contraceptive use (OR=0.76; 95% CI=0.54, 0.97) were factors significantly associated with hot flushes. No significant relationship was found for marital status, occupation, economical situation, smoking, parity, abortion, body mass index, and age at first live birth.

**Table 4:** Adjusted odds ratio (OR) for hot flushes according to socio-demographic characteristic, smoking, reproductive history and body mass index (No=1397)

**Variable**	**Adjusted OR (95% CI)**	**p value**
	**Mean±SD (n=740)**	
Marital status		0.48
Married	0.61 (0.16±2.35)	
Single	1.00	
Education		0.006
Less than elementary level	0.70 (0.54±0.90)	
At least elementary level	1.00	
Occupation		0.94
House wife	0.99 (0.68±1.42)	
Working	1.00	
Economic situation		0.96
With saving	0.99 (0.72±1.37)	
With debts	1.00	
Smoking		0.22
Smoker	0.44 (0.16±1.65)	
Non-smoker	1.00	
Age at menarche		0.048
<13 years	0.76 (0.59±0.99)	
≥13 years	1.00	
Parity		0.74
Childless	1.13 (0.54±2.36)	
At least one child	1.00	
Abortion		0.48
No abort	1.09 (0.86±1.38)	
At least one abort	1.00	
Oral contraceptive used		0.03
Yes	0.73 (0.54±0.97)	
No	1.00	
Body mass index		1.00
≤25 kg/m^2^	1.00 (0.75±1.34)	
>25kg/m^2^	1.00	
Age at first live birth		0.19
<20 years	1.17 (0.92±1.49)	
≥20 years	1.00	

## Discussion

In this study, the mean age in menopause was 47.7 years. A number of surveys of Asian population showed that there is a difference in age at menopause between developed and developing countries. The mean age at menopause in Asia is lower than in developed countries.[10][11]

In our study, the mean age of normal menopause was similar to that reported by women from Shiraz (47.8 years)[12] and Pakistan,[13] however, it was lower than that of Iran (49.6 years)[14] and the USA (51.4 years),[10] but it was higher than Egypt (46.7 years).[15] The possible explanations for the relatively lower mean age in menopause were the differences in the definition of menopause, population sample and the survey method. Hormone replacement therapy in the perimenopausal period could be an important factor of delayed menopause in developed countries.

It was shown that menopause occurs earlier in women who are smoking,[16] but we did not find any significant difference for age in menopause, oral contraceptive use and smoking. The use of oral contraceptive and smoking in women aged 45-63 years was low. Some studies reported that menopause occur earlier in nulliparous women, [17][18] while similar associations were found in our study.

The age in menopause was self-reported since it would not be easy for the elderly women to recall their age in menopause. The misclassification bias is possible in the classification of the groups for age in menopause, but it is unlikely that any bias was different in the strata of marital status, education, parity, occupation or the other considered factors. Weight and height were measured directly and consequently the probable information bias due to self reports was minimized.

In this study, the most prevalent symptoms were irritabilitis, joint pains, and backache. These results are in line with many Asian studies indicating that these symptoms particularly joint pains are the most common symptoms.[19][20] Psychological (irritabilities) and somatic (joint pains, backache) symptoms are not entirely related to menopause. Such symptoms could be due to several factors such as health problems associated with aging women, midlife crises, and other non-menopausal factors experienced by 45-63 years old women.

Almost every woman will experience hot flushes during her lifetime.[21] In one study, more than 60 respondents experienced hot flushes.[22] This is in line with our findings too. The experience of hot flushes at the time of menopause in our study was lower than western countries [23] and higher than Asian countries reporting a low prevalence of hot flushes (9.8%-38.5%).[24][25][26][27][28] Hot flushes may be associated with anxiety and depressive symptoms in Asian women with low vasomotor symptoms.[29][30] The exact pathophysiology of hot flushes is not clear [21] and may be influenced by diet [31] and age at menarche.[32] However, some studies suggested that Asian diet with higher level of phytoestrogens causes a low prevalence of hot flushes in Asian women.[33] Hot flushes are multi-factorial but result primarily from the estrogen withdrawal in menopausal women. Pattern of hot flushes may change over time. It typically lasts for 0.5 to 5.0 years after normal menopause. Small percentage of postmenopausal women may experience hot flushes more frequently for as long as 15 years.[34][35] Estrogen virtually eliminates hot flushes but its mechanism of action is not known.[36] Hot flushes was significantly lower for menopausal women using oral contraceptive pills than those who did not which is consistent with the study of Ayranic et al.[37] Staropoli et al.[22] reporting that hot flushes are not associated with demographic, reproductive or behavior characteristics. Our finding is consistent with this study. In the same study, hot flushes were associated with cigarette smoking, while in our study, there was not any relationship between hot flushes and cigarette smoking. This may be due to a low number of smokers in our study.

Our study is a retrospective design but prospective and longitudinal studies may provide stronger evidences on this association. However, prospective trials have their own weaknesses too. Also, there is a possibility of recall bias in experience of hot flushes, but the information about menopausal symptoms during previous two weeks of interview was obtained using a standard questionnaire to minimize the misclassification bias in menopausal symptom assessment.

Almost all of the subjects reported that they did not use any hormone replacement therapy and calcium supplement concerning menopause and about 72% did not consult a doctor and ignored their symptoms. In addition, the mean age at menopause was 47.7 years. So it could be beneficial to provide evidences to manage menopausal women in Primary Health Care centers as a cost effective method. Emphasis should be placed on practical education of women to raise their knowledge on menopausal symptoms and ways of increasing their understanding on midlife experiences.
